# Antagonistas do Sistema Renina-Angiotensina e Betabloqueadores na Prevenção da Cardiotoxicidade por Antraciclinas: Revisão Sistemática e Metanálise

**DOI:** 10.36660/abc.20220298

**Published:** 2023-05-17

**Authors:** Monica Samuel Avila, Suellen Rodrigues Rangel Siqueira, Lucas Waldeck, Silvia Moreira Ayub-Ferreira, Richard Takx, Marcio Sommer Bittencourt, Edimar Alcides Bocchi

**Affiliations:** 1 Hospital das Clínicas Faculdade de Medicina Universidade de São Paulo São Paulo SP Brasil Departamento de Insuficiência Cardíaca – Instituto do Coração (InCor) do Hospital das Clínicas da Faculdade de Medicina da Universidade de São Paulo, São Paulo, SP – Brasil; 2 Departmento de Radiologia University Medical Center Utrecht Utrecht Holanda Departmento de Radiologia – University Medical Center Utrecht, Utrecht – Holanda; 3 Centro de Pesquisas Clínicas e Epidemiológicas Hospital Universitário Universidade de São Paulo São Paulo SP Brasil Centro de Pesquisas Clínicas e Epidemiológicas – Hospital Universitário – Universidade de São Paulo, São Paulo, SP – Brasil

**Keywords:** Tratamento farmacológico, Insuficiência Cardíaca, Inibidores da Enzima Conversora de Angiotensina, Antagonistas de Receptores de Mineralocorticoides, Antraciclinas

## Abstract

**Fundamento:**

As evidências que embasam o uso de inibidores do sistema-renina-angiotensina aldosterona (SRAA) e betabloqueadores para prevenção de cardiomiopatia induzida por antraciclinas são controversas.

**Objetivo:**

Realizamos uma metanálise para avaliar a eficácia desses medicamentos na prevenção da cardiotoxicidade.

**Métodos:**

A metanálise incluiu estudos prospectivos e randomizados com adultos submetidos à quimioterapia com antraciclina e comparou o uso de terapias SRAA ou betabloqueadores versus placebo com seguimento de 6 a 18 meses. O desfecho primário foi alteração da fração de ejeção do ventrículo esquerdo (FEVE) durante a quimioterapia. Os desfechos secundários foram: a incidência de insuficiência cardíaca, mortalidade por todas as causas e alterações na medida do diâmetro diastólico final. A avaliação da heterogeneidade foi realizada por estratificação e meta-regressão. O nível de significância adotado foi p < 0,05.

**Resultados:**

A busca resultou em 17 estudos, totalizando 1.530 pacientes. A variação (delta) da FEVE foi avaliada em 14 estudos. A terapia neuro-hormonal foi associada a um menor delta na FEVE pré-terapia versus pós-terapia (diferença média ponderada 4,42 [intervalo de confiança de 95% 2,3 a 6,6]) e maior FEVE final (p < 0,001). O tratamento resultou em menor incidência de insuficiência cardíaca (risk ratio 0,45 [intervalo de confiança de 95% 0,3 a 0,7]). Não houve efeito na mortalidade (p = 0,3). Para a análise da FEVE, foi documentada heterogeneidade substancial, não explicada pelas variáveis exploradas no estudo.

**Conclusão:**

O uso de inibidores do SRAA e betabloqueadores para prevenção da cardiotoxicidade induzida por antraciclinas foi associado a redução menos pronunciada da FEVE, maior FEVE final e menor incidência de insuficiência cardíaca. Não foram observadas alterações na mortalidade. (CRD PROSPERO 42019133615)

## Introdução

O câncer é uma das principais causas de morte no mundo.^1^ A incidência de sobrevida em pacientes com câncer melhorou nos últimos anos, principalmente devido ao sucesso do tratamento quimioterápico.^2^ No entanto, o prognóstico desses pacientes permanece limitado devido às complicações do tratamento, como a cardiotoxicidade das antraciclinas, resultando em insuficiência cardíaca.^3^

Várias estratégias para a prevenção primária da cardiotoxicidade induzida por antraciclinas têm sido propostas. A prevenção envolve abordagens que minimizam a exposição da droga, resultando em menor risco de cardiotoxicidade potencial e na decisão de iniciar drogas cardioprotetoras. O uso de drogas cardiovasculares, como inibidores da enzima conversora de angiotensina (IECA), bloqueador do receptor de angiotensina (BRA), antagonista do receptor de mineralocorticoide (ARM) e betabloqueadores é baseado em poucos ensaios clínicos com resultados controversos. O uso de tratamento preventivo com IECA, BRA, ARM ou terapia com betabloqueadores em pacientes em quimioterapia com antraciclinas com baixo risco cardiovascular basal permanece incerto, e nenhuma recomendação pode ser feita atualmente.^4^

Existem poucas metanálises publicadas avaliando terapias com antagonistas neuro-hormonais na prevenção da cardiotoxicidade. Alguns estudos incluíram populações pediátricas^5,6^ e outras intervenções como estatinas, dexrazoxano ou N-acetilcisteína,^7-10^ enquanto outros estudos incluíram apenas betabloqueadores^11-15^ ou apenas antagonistas do sistema renina-angiotensina-aldosterona (SRAA).^16,17^ Recentemente, Vaduganathan et al. publicaram uma metanálise avaliando IECA, BRA, ARM e betabloqueadores na prevenção da cardiotoxicidade relacionada à quimioterapia, incluindo antraciclina e trastuzumabe.^18^ Conforme estabelecido, os mecanismos cardiotóxicos das antraciclinas e terapias anti-HER2 são distintos, o que poderia ser um fator de confusão para o real impacto da prevenção da cardiotoxicidade por antagonistas neuro-hormonais.

Diante de evidências controversas que apoiam o uso de inibidores do sistema da angiotensina e betabloqueadores apenas para prevenção primária da cardiotoxicidade induzida por antraciclinas, realizamos uma revisão sistemática e metanálise para avaliar a eficácia desses agentes como drogas profiláticas para início precoce de cardiotoxicidade.

## Métodos

### Busca da literatura

Seguimos a declaração Preferred Reporting Items for Systematic Reviews and Meta-Analyses (PRISMA), e a lista de verificação PRISMA é apresentada no Material Suplementar.^19^ Nosso protocolo de estudo pré-especificado foi registrado no International Prospective Register of Systematic Reviews (PROSPERO CRD 42019133615). Pesquisamos sistematicamente PubMed, EMBASE, ClinicalTrials.Gov e Cochrane Central Register of Controlled Trials para ensaios controlados randomizados de drogas cardioprotetoras, como betabloqueadores, IECA, BRA e ARM, em pacientes sob quimioterapia com antraciclina para avaliar a eficácia desses medicamentos na prevenção da cardiotoxicidade. A lista de termos utilizados na busca é apresentada no Material Suplementar. Limitamos a busca a artigos em inglês. Além disso, pesquisamos as referências de todos os artigos identificados. Incluímos todos os ensaios controlados randomizados usando medicamentos cardioprotetores no braço ativo (IECA, betabloqueador, BRA ou ARM) comparados com placebo ou tratamento usual, com seguimento de 6 a 18 meses, que relataram a função cardíaca avaliada por ecocardiograma ou ressonância magnética cardíaca, diâmetros cardíacos e/ou desfechos clínicos (morte, insuficiência cardíaca). Excluímos resumos, estudos com períodos de seguimento mais curtos, população pediátrica, estudos sem braço controle e estudos não randomizados. Nenhum paciente foi incluído, e todos os dados do estudo são anônimos; portanto, não foi necessária aprovação do comitê de ética ou conselho de revisão institucional.

### Extração de dados e desfechos

Dois investigadores (M.S.A. e S.R.R.S.) extraíram dados de forma independente usando um formulário padronizado, incluindo características do estudo (desenho, critérios de inclusão e exclusão), características da intervenção (medicamento cardioprotetor), características do paciente (idade, sexo, fatores de risco cardíaco, malignidade) e desfechos. Para os desfechos, definimos a priori o desfecho primário de alteração (delta) na fração de ejeção do ventrículo esquerdo (FEVE) desde a linha de base até o final do estudo. Desfechos secundários definidos a priori incluíram mortes por todas as causas, insuficiência cardíaca e alterações (delta) na medição do diâmetro diastólico final por ecocardiografia.

### Síntese de dados

Realizamos uma síntese narrativa dos achados dos estudos incluídos, que compreendia descrição do tipo de tratamento, características da população, desfechos e conteúdo da intervenção. Fornecemos resumos dos efeitos da intervenção para cada estudo, calculando as *odds ratios* para resultados dicotômicos e diferenças médias ponderadas para os desfechos contínuos. Para estudos que não relataram as diferenças longitudinais nas mudanças dos parâmetros ecocardiográficos ao longo do tempo, usamos as diferenças relatadas com desvios-padrão, erros-padrão ou intervalos de confiança. Para os estudos que não relataram nenhuma das medidas de dispersão para a alteração (delta), os erros-padrão foram derivados do desvio-padrão das pré- e pós-medidas, inserindo os valores das correlações entre o pré-teste e o pós-teste, com base na correlação derivada de outros estudos nos quais tivemos acesso a dados individuais do nível do paciente para derivar os coeficientes. A descrição dos resultados dos ensaios e os critérios de inclusão/exclusão estão detalhados no Material Suplementar.

### Estratificação e análise de sensibilidade

Imaginamos que um grande número de estudos com diferentes desfechos e intervenções pudesse resultar em uma alta heterogeneidade, assim, realizamos todas as análises usando modelos de efeitos aleatórios. Além disso, quando a heterogeneidade medida pelo teste χ^2^ e o I^2^ era superior a 50%, indicando heterogeneidade substancial, realizávamos análises adicionais de acordo com a qualidade do estudo, data do estudo, tipo de medicamento usado para tratamento, dose de antraciclina e características dos pacientes incluídos no estudo. Esta análise foi realizada usando metanálises estratificadas para preditores categóricos e meta-regressão para preditores contínuos. Também avaliamos evidências de viés de publicação usando gráficos de funil. A análise estatística foi realizada no Stata 17.0 (StataCorp, Estados Unidos), e o nível de significância foi definido como p < 0,05.

### Avaliação de qualidade

Dois investigadores (M.S.A. e S.R.R.S.) avaliaram independentemente a qualidade dos estudos usando a ferramenta da Cochrane Collaboration para avaliar o risco de viés em ensaios randomizados.^20^ As discordâncias foram resolvidas por consenso.

### Qualidade dos ensaios

Dois investigadores (M.S.A. e S.R.R.S.) avaliaram a qualidade dos estudos usando a Ferramenta de Risco de Viés Cochrane. Em particular, a avaliação considerou: geração de sequência aleatória, ocultação de alocação, ocultação de participantes e da equipe, ocultação de desfecho e avaliação, avaliação de dados e outros vieses. A qualidade do estudo é detalhada no Material Suplementar.

## Resultados

**Figura 1:**
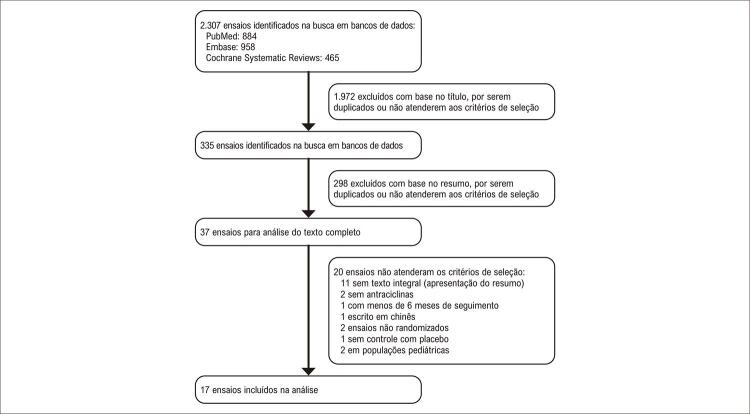
– Processo de seleção dos ensaios para a revisão sistemática.

A busca sistemática resultou em 355 artigos potencialmente relevantes. Após a remoção dos ensaios que não atendiam aos critérios de inclusão, com período de seguimento inferior a 6 meses, ensaios não randomizados, sem controle com placebo e população pediátrica, foram incluídos 17 ensaios na análise. O diagrama para a seleção dos estudos é mostrado na Figura 1. Os estudos incluídos de 2006 a 2018 com 1.530 pacientes tiveram critérios de inclusão semelhantes, exceto pelo tipo de câncer, embora o câncer de mama tenha sido a doença mais frequente. As características dos estudos incluídos e os dados demográficos basais dos pacientes estão presentes na Tabela 1. Sete estudos eram duplo-cegos, enquanto 3 eram simples-cegos e 7 não eram cegos. O seguimento foi de 6 meses em 13 ensaios clínicos e de 12 meses em 3 ensaios. Dez dos estudos testaram a influência de betabloqueadores (carvedilol, metoprolol ou nebivolol); dois testaram IECA (enalapril); um testou BRA (telmisartan ou candesartan); um avaliou o antagonismo da aldosterona (espironolactona); dois analisaram a associação de IECA e betabloqueador, e um testou a associação de BRA e betabloqueador.

**Tabela 1:** – Características basais dos estudos randomizados. Estudos que utilizaram mais de um medicamento cardioprotetor foram desmembrados para melhor análise. Todos os estudos adotaram significância estatística de 5%

Estudo	Ano	MCPT	FEM, CTL n^**o**^	FEM, MED n^**o**^	Idade, CTL (anos)+	Idade, MED (anos)+	Pts, CTL n^**o**^	Pts, CTL n^**o**^	Malignidade	ANT	HPTN clt n^**o**^ (%)	HPTN, MED n^**o**^ (%)	DM clt n^**o**^ (%)	DM, MED n^**o**^ (%)	HLP, CTL n^**o**^ (%)	HLP, MED n^**o**^ (%)	Fumante, CTL n^**o**^ (%)	Fumante, MED n^**o**^ (%)	Radiação, CTL n^**o**^ (%)	Radiação, MED n^**o**^ (%)	Seguimento (meses)
Kalay^21^	2006	CVDL	21	22	49	46,8	25	25	LIN, CM	DOX, EPI	NA	NA	NA	NA	NA	NA	NA	NA	0	0	6
Cardinale^22^	2006	ENLP	39	33	44	47	58	56	LA, CM, SE, LH, MLM, LNH	EPI, IDA, DAU	4 (7)	3 (5)	1 (2)	1 (2)	2 (3)	2 (4)	NA	NA	18 (31)	19 (34)	12
Georgakopoulos^26^	2010	MTPL	19	20	49,1	51	40	42	LH, LNH	DOX	6 (15)	10 (24)	6 (15)	10 (24)	10 (25)	14 (33)	16 (40)	17 (40)	9 (23)	8 (19)	12
Georgakopoulos	2010	ENLP	19	21	49,1	47,4	40	43	LH, LNH	DOX	6 (15)	14 (33)	6 (15)	3 (7)	10 (25)	11 (26)	16 (40)	20 (46)	9 (23)	9 (21)	12
Salehi^27^	2011	CVDL*	14	32	43,5	43,5	22	44	LIN, CM	DOX, EPI	NA	NA	NA	NA	NA	NA	NA	NA	0	0	4
Dessì^28^	2011	TMST	18	19	53	52,9	24	25	LNH, CM, outras	EPI	0	0	0	0	NA	NA	NA	NA	0	0	12
Kaya^29^	2013	NBVL	18	27	50,5	51,4	18	27	CM	DOX, EPI	4 (22)	6 (22)	2 (11)	2 (7)	NA	NA	NA	NA	5 (28)	7 (26)	6
Bosch/OVERCOME^30^	2013	ENLP, CVDL	21	18	50,9	49,7	45	45	LA, LH, LNH	IDA, DAU	8 (18)	6 (13)	3 (7)	7 (16)	1 (2)	3 (7)	4 (9)	13 (29)	4 (9)	12 (27)	6
Elitok^31^	2014	CVDL	40	40	52,9	54,3	40	40	CM	DOX	0	0	0	0	NA	NA	NA	NA	NA	NA	6
Akpek^32^	2014	ESPL	40	43	50,6	50	40	43	CM	DOX, EPI	0	0	NA	NA	NA	NA	NA	NA	NA	NA	6
Gulati/PRADA^23^	2016	CDST, MTPL	32	32	50,8	50	32	32	CM	EPI	0	1 (3)	0	0	NA	NA	7 (22)	6 (20)	23 (72)	18 (60)	6
Gulati/PRADA	2016	CDST	32	33	50,8	51,7	32	33	CM	EPI	0	5 (16)	0	1 (3)	NA	NA	7 (22)	7 (22)	23 (72)	19 (60)	6
Gulati/PRADA	2016	MTPL	32	32	50,8	50,5	32	32	CM	EPI	0	2 (6)	0	1 (3)	NA	NA	7 (22)	5 (16)	23 (72)	22 (69)	6
Jhorawat^33^	2016	CVDL	9	4	38,7	43,89	27	27	LNH, LH, LA	DOX	NA	NA	0	0	NA	NA	NA	NA	NA	NA	6
Beheshti^34^	2016	CVDL	40	30	39,9	42	40	30	CM	DOX	0	0	0	0	NA	NA	NA	NA	0	0	6
Nabati^35^	2017	CVDL	45	46	47,1	47,5	45	46	CM	DOX	5 (12)	11 (27)	5 (12)	3 (7)	NA	NA	NA	NA	0	0	6
Janbabai^36^	2017	ENLP	31	33	47	47,7	35	34	CM, LH, outras	DOX	4 (11)	6 (18)	5 (14)	3 (9)	3 (9)	4 (12)	NA	NA	0	0	6
Abuosa^37^	2018	CVDL**	29	83	40,4	46,1	38	116	CM, LIN outras	DOX	4 (10)	14 (12)	6 (16)	21 (18)	2 (5)	6 (5)	NA	NA	NA	NA	6
Cochera ^38^	2018	NBVL	30	30	52	53	30	30	CM	DOX	0	0	0	0	3 (10)	4 (13)	3 (10)	2 (6)	0	0	6
Avila/CECCY^24^	2018	CVDL	96	96	52,9	50,8	96	96	CM	DOX	9 (9)	3 (3)	5 (5)	4 (4)	2 (2)	6 (6)	26 (27)	24 (25)	0	0	6

ANT: antraciclina; CDST: candesartan; CM: câncer de mama; CTL: grupo controle; CVDL: carvedilol; DAU: daunorrubicina; DM: diabetes mellitus; DOX: doxorrubicina; ENLP: enalapril; EPI: epirrubicina; ESPL: espironolactona; FEM: pacientes do sexo feminino; HLP: hiperlipidemia; HPTN: hipertensão; IDA: idarrubicina; LA: leucemia aguda; LH: linfoma de Hodgkin; LIN: linfoma; LNH: linfoma não Hodgkin; MCPT: medicamento cardioprotetor; MED: grupo que recebeu o tratamento medicamentoso; MLM: mieloma; MTPL: metoprolol; NA: não aplicável; No: número de pacientes; NBVL: nebivolol; Outras: endométrio; glândula salivar; ovário; câncer de pulmão; tumor de Wilms; Pts: pacientes; SE: sarcoma de Ewing; TMST: telmisartana. + A idade foi expressa em mediana. * O estudo testou as doses de 12,5 mg e 25 mg.

Todos os 17 estudos avaliaram a função ventricular esquerda, e 10 estudos analisaram o diâmetro diastólico final do ventrículo esquerdo (DDFVE) para a detecção de cardiotoxicidade por ecocardiografia. A doxorrubicina foi a quimioterapia com antraciclina mais frequentemente incluída nos ensaios, e a dose cumulativa mediana (faixa interquartil) foi de 241 (240 a 369) mg/m^2^ no grupo placebo e 286 (254,2 a 383) no grupo dos medicamentos cardioprotetores.

### Alteração absoluta na fração de ejeção do ventrículo esquerdo

Catorze estudos analisaram o delta da fração de ejeção. Os valores de FEVE e DDVE desde a linha de base até o final dos estudos estão resumidos na Tabela 2. Os resultados agrupados mostraram que os pacientes que receberam betabloqueadores e bloqueadores do SRAA apresentaram alterações menos acentuados na FEVE do que o grupo controle (diferença média ponderada do delta da FEVE: 4,42; intervalo de confiança de 95% 2,27 a 6,57; p = 0,0001; Figura Central). No entanto, foi observada heterogeneidade significativa, mesmo após a estratificação por medicamento usado no tratamento (I^2^ = 92,7%), embora os tamanhos de efeito fossem comparáveis para todos os medicamentos. Metarregressões adicionais usando idade, dose cumulativa de antraciclinas ou ano do estudo não foram capazes de identificar qualquer fator associado à heterogeneidade.

**Tabela 2:** – Alterações da FEVE e DDFVE. Estudos que utilizaram mais de um medicamento cardioprotetor foram desmembrados para melhor análise

Estudo	Ano	DCANT, CTL (mg/m^**2**^)	DCANT, MED (mg/m^**2**^)	FEVE basal, CTL (%)	FEVE basal, MED (%)	FEVE final do estudo, CTL (%)	FEVE final do estudo, MED (%)	DDFVE basal, CTL (mm)	DDFVE basal, MED (mm)	DDFVE final do estudo, CTL (mm)	DDFVE final do estudo, MED (mm)
Kalay^21^	2006	513,6	525,3	69,7 + 7,3	70,6 + 8	52,3 + 14	69,7 + 6	45,6 + 5	47,6 + 5,6	50,9 + 5,6	47,4 + 3,7
Cardinale^22^	2006	338 + 167	332 + 191	62,8 + 3,4	61,9 + 2,9	51,9 + 7,9	61,3 + 3,9	NA	NA	NA	NA
Georgakopoulos^26^	2010	386,4 + 5,7	387,5 + 6,8	67,6 + 7,1	65,7 + 5	66,6 + 6,7	63,3 + 7,4	48 + 6	47 + 5	48 + 5	49 + 4
Georgakopoulos	2010	386,4 + 5,7	373,1 + 6,3	67,6 + 7,1	65,2 + 7,1	66,6 + 6,7	63,9 + 7,5	48 + 6	49 + 4	48 + 5	50 + 5
Salehi^27^Carvedilol 12.5 mg	2011	540,2 + 31,1	531,5 + 29,9	58,56 + 3,62	60,5 + 5,07	53,9 + 3,8	53,1 + 7,76	41,3 + 0,6	41,7 + 0,39	45,6 + 0,57	45 + 0,46
Salehi Carvedilol 25 mg	2011	540,2 + 31,1	521,14± 38,97	58,56 + 3,62	61 + 7,06	53,9 + 3,8	56,8 + 6,2	41,3 + 0,6	39,3 + 0,34	45,6 + 0,57	40,9 + 0,37
Dessì^28^	2011	400	400	66 + 5	66 + 7	65+ 7	68 + 4	NA	NA	NA	NA
Kaya^29^	2013	235 + 48	527 + 29	66,6 + 5	65,6 + 4,8	57,5 + 5,6	66,6 + 5,5	47,2 + 3,8	47 + 4,4	52 + 4,6	47,1 + 4
Bosch Overcome^30^	2013	241 + 162	290 + 189	62,59 + 5,38	61,67 + 5,11	59 + 6	62 + 5	NA	NA	NA	NA
Elitok^31^	2014	523,3	535,6	65 + 4,5	66 + 6,1	63,3 + 4,8	64,1 + 5,1	44,3 + 3,1	45 + 14,2	44,1 + 4,1	44,6 + 3,2
Akpek^32^	2014	394,2	430,2	67,7 + 6,3	67 + 6,1	53,6 + 6,8	65,7 + 7,4	46 + 5	46 + 4	52 + 4	49 + 4
Gulati PRADA^23^candesartam +metoprolol	2016	301,3 + 75,57	297,3 + 72,5	63,6 + 4,1	62,2 + 4,4	60,3	61,1	NA	NA	NA	NA
Gulati PRADA canndesartan	2016	301,3 + 71,57	297,5 + 71,8	63,6 + 4,1	62,3 + 5,3	60,3	61,63	NA	NA	NA	NA
Gulati PRADA metoprolol	2016	301,3 + 75,57	301,3 + 72,5	63,6 + 4,1	63,5 + 5,0	60,3	60,8	NA	NA	NA	NA
Jhorawat^33^	2016	252,6 + 77,82	267,3 + 76,1	67,56+ 5,98	63,19+ 7,22	60,82+ 11,28	63,88+ 8,56	47,24+ 5,13	46,35+ 7,71	48,5 + 5,75	47,95 + 5,28
Beheshti^34^	2016	240	240	59,41+ 4,20	61,31+ 3,21	59,30 + 4,29	61,06 + 3,39	NA	NA	NA	NA
Nabati^35^	2017	359,9 + 27,1	348,5 + 34,8	61,13 + 4,97	58,72 + 4,69	51,67 + 6,01	57,44 + 7,52	NA	NA	NA	NA
Janbabai^36^	2017	266,6 + 21,7	363,3 + 34,8	59,61 + 5,70	59,39 + 6,95	46,31 + 7,04	59,93 + 7,83	NA	NA	NA	NA
Abuosa^37^ Carvedilol 6.25 mg	2018	265,6 + 98,5	252 + 65	62,0 + 4,6	61,4 + 3,9	58,2 + 6,6	61,4 + 3,9	45,3 + 5,3	46,0 + 5,1	45,9 + 7,5	46,8 + 4,0
Abuosa Carvedilol 12.5 mg	2018	265,6 + 98,5	282 + 78	62,0 + 4,6	60,0 + 4,2	58,2 + 6,6	60,0 + 4,1	45,3 + 5,3	44,8 + 4,3	45,9 + 7,5	46,0 + 3,7
Abuosa Carvedilol 25 mg	2018	265,6 + 98,5	261 + 101	62,0 + 4,6	60,5 + 4,2	58,2 + 6,6	60,4 + 4,2	45,3 + 5,3	44,6 + 6,3	45,9 + 7,5	45,5 + 5,3
Cochera^38^	2018	519 + 9	521 + 6	61+ 2	62+ 4	60 + 3	61 + 3	44,8 + 4,2	45,1+ 4,2	46,1 + 3,5	46,2 + 2,9
Avila CECCY^24^	2018	240	240	65,2 + 3,6	64,8 + 4,7	63,9 + 5,2	63,9 + 3,8	44,9 + 3,6	44,1 + 3,3	46,4 + 4,0	45,2 + 3,2

CTL: grupo controle; DCANT: dose cumulativa de antraciclinas; DDFVE: diâmetro diastólico final do ventrículo esquerdo; FEVE: fração de ejeção do ventrículo esquerdo; IC: insuficiência cardíaca; MED: grupo que recebeu o tratamento medicamentoso; NA: não se aplica; No: número de pacientes. + A avaliação da FEVE foi realizada por ressonância magnética cardíaca. ** O estudo testou as doses de 6,25 mg, 12,5 mg e 25 mg.

**Figura Central:**
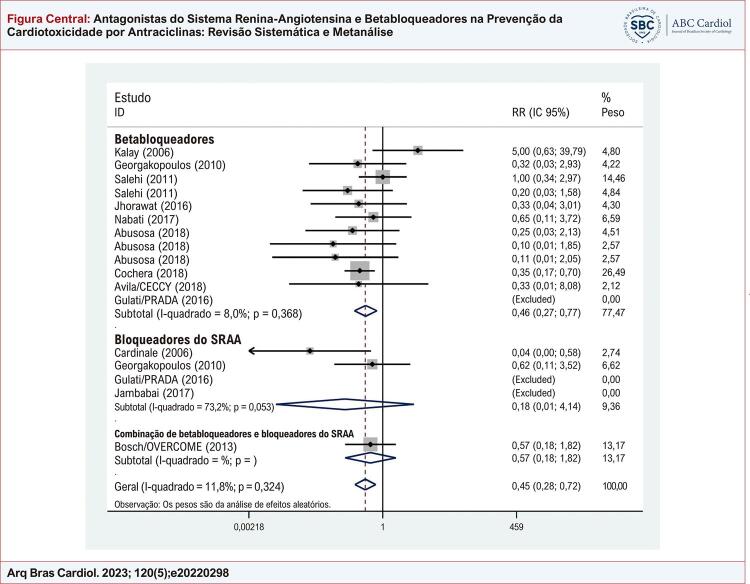
: Antagonistas do Sistema Renina-Angiotensina e Betabloqueadores na Prevenção da Cardiotoxicidade por Antraciclinas: Revisão Sistemática e Metanálise

Impacto dos medicamentos cardioprotetores na fração de ejeção do ventrículo esquerdo. IC: intervalo de confiança; RR: risk ratio; SRAA: sistema renina-angiotensina-aldosterona.

### Insuficiência cardíaca e mortalidade

**Figura 2:**
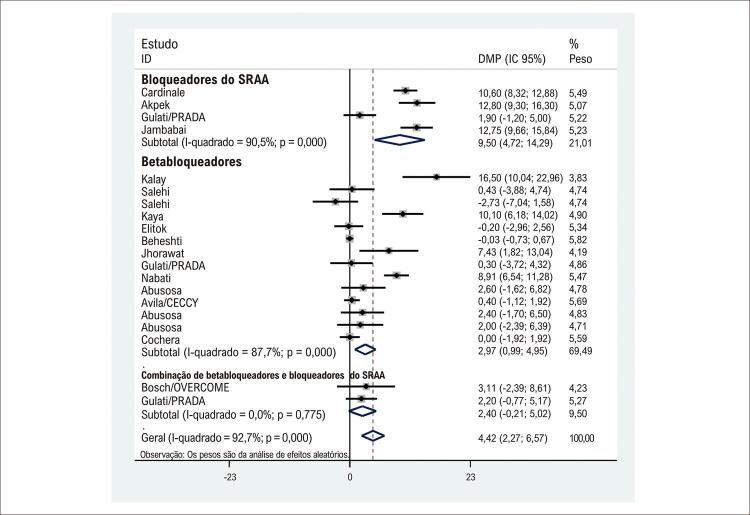
– Impacto dos medicamentos cardioprotetores na ocorrência de insuficiência cardíaca. DMP: diferença média ponderada; IC: intervalo de confiança; RAAS: sistema renina-angiotensina-aldosterona.

Doze estudos relataram a influência de drogas neuro-hormonais e betabloqueadores na incidência de insuficiência cardíaca e onze na morte. No entanto, após agrupar os resultados dos doze estudos, a presença de medicamentos cardioprotetores foi associada a menos sintomas de insuficiência cardíaca durante e após o uso de antraciclina (*risk ratio* 0,45; intervalo de confiança de 95% 0,28 a 0,72; p = 0,32; Figura 2). A heterogeneidade entre os estudos não foi significativa (I^2^ = 11,18%), e nenhum viés de publicação potencial foi identificado. Os números absolutos de insuficiência cardíaca e morte são relatados no Material Suplementar.

## Discussão

A presente metanálise analisou os efeitos protetores dos inibidores do SRAA e dos betabloqueadores contra a cardiotoxicidade induzida por antraciclinas. Selecionamos 17 ensaios randomizados e encontramos um benefício dos agentes cardioprotetores nas alterações da FEVE e nos sintomas de insuficiência cardíaca. A terapia neuro-hormonal foi associada a um menor delta na FEVE e menos sintomas de insuficiência cardíaca, e não houve efeito na mortalidade. Apesar do impacto positivo das drogas neuro-hormonais, encontramos alta heterogeneidade entre os estudos; portanto, a interpretação desses achados precisa ser contextualizada, e um potencial viés de publicação deve ser considerado.

O campo da cardio-oncologia tem sido amplamente estudado nos últimos 15 anos. Kalay et al.^21^ mostraram, em 2006, que o uso de carvedilol pode prevenir a diminuição da fração de ejeção e o aumento dos diâmetros do ventrículo esquerdo em pacientes em uso de antraciclinas, sem alterar significativamente a mortalidade. Em outro estudo importante, Cardinale et al.^22^ mostraram que o uso de IECA poderia reduzir a elevação do diâmetro sistólico do ventrículo esquerdo e prevenir a cardiotoxicidade em pacientes que apresentaram maiores alterações de troponina após o ciclo quimioterápico.

Mais recentemente, o ensaio PRADA, um estudo randomizado e controlado por placebo, avaliou o uso de candesartana, metoprolol e o uso combinado de ambas as drogas na prevenção primária da cardiotoxicidade da antraciclina. O estudo observou benefício apenas com a candesartana, demonstrando menor volume extracelular avaliado pela ressonância magnética e redução atenuada da FEVE.^23^ O ensaio randomizado mais recentemente publicado, o CECCY Trial, foi um estudo randomizado de centro único que testou o carvedilol como protetor cardíaco em pacientes com câncer de mama submetidas a quimioterapia com antraciclinas. Não mostrou diferença significativa na disfunção ventricular, mas mostrou um benefício no diâmetro diastólico do ventrículo esquerdo e troponina no grupo carvedilol.^24^

A análise do desfecho de insuficiência cardíaca realizada individualmente em cada estudo não apresentou diferença estatística. Porém, quando analisamos a população total de todos os estudos, observamos melhor evolução no grupo em uso de betabloqueadores e inibidores do SRAA, com resultados significativos.

A heterogeneidade difere entre os desfechos analisados. Observamos heterogeneidade significativa na avaliação do delta da fração de ejeção, o que potencialmente reflete a variação na população dos estudos devido a diferenças na terapia cardioprotetora, malignidade e doses de antraciclinas. Quanto à avaliação do desfecho clínico, observamos baixa heterogeneidade.

Algumas metanálises avaliaram o impacto da terapia neuro-hormonal na cardiotoxicidade induzida por antraciclinas. Kheiri et al. avaliaram o impacto do carvedilol na prevenção da cardiotoxicidade induzida por ANT e demonstraram um possível benefício atenuando a diminuição da FEVE. No entanto, este estudo não incluiu inibidores de RAAS.^11^ Recentemente Caspani et al. publicaram uma metanálise que avaliou as terapias neuro-hormonais nesse cenário, incluindo um número menor de ensaios e tamanho amostral. Encontraram benefício na prevenção da redução da FEVE no braço que recebeu o medicamento, mas não encontraram impacto do medicamento cardioprotetor na insuficiência cardíaca.^25^ Vaduganathan et al. publicaram uma metanálise avaliando IECA, BRA, ARM e betabloqueadores na prevenção da cardiotoxicidade relacionada à quimioterapia, incluindo antraciclina e trastuzumabe. Os autores concluíram que as terapias neuro-hormonais tiveram um impacto positivo na redução do declínio da função ventricular esquerda com alta heterogeneidade, o que é consistente com nossa análise. Apesar disso, a inclusão de um estudo com trastuzumabe pode ser um fator de confusão, já que o mecanismo de cardiotoxicidade é diferente das antraciclinas.^18^

Nossos resultados revelam a necessidade de estudos com populações maiores, com maior potencial para mostrar o real benefício dos medicamentos cardioprotetores na cardiotoxicidade.

### Limitações do estudo

Nossa metanálise tem várias limitações importantes. A maioria dos estudos incluídos avaliou a FEVE usando ecocardiografia padrão, e poucos incluíram a estrutura do ventrículo esquerdo. Alterações na FEVE são uma medida muito heterogênea, e não foi relatada a variabilidade interobservador em todos os estudos. Em relação ao desfecho de insuficiência cardíaca, há dados ausentes em alguns artigos, o que pode comprometer os resultados para esse desfecho. Em relação à dose de antraciclina, alguns artigos relataram a dose total de antraciclina e não relataram a dose em mg/m^2^. Além disso, como os estudos incluíram diferentes antraciclinas, as doses foram diferentes entre os ensaios. Os tamanhos de amostra limitados em alguns estudos e dados ausentes sobre fatores de risco cardiovascular podem impedir análises de subgrupos por risco cardiovascular.

## Conclusão

Concluímos que antagonistas do SRAA e betabloqueadores para prevenção da cardiotoxicidade induzida por antraciclinas foram associados à redução menos pronunciada da FEVE, maior FEVE final e menor incidência de insuficiência cardíaca. Não foram observadas alterações na mortalidade. Foi observada heterogeneidade significativa entre os estudos na avaliação do delta da fração de ejeção, o que potencialmente reflete a variação na população dos estudos. É necessário realizar mais estudos com populações maiores, com resultados consistentes e significativos que demonstrem o benefício dos medicamentos cardioprotetores na cardiotoxicidade.

## *Material suplementar

Para informação adicional, por favor, clique aqui


