# Supplementary material for: Antagonistas do Sistema Renina-Angiotensina e Betabloqueadores na Prevenção da Cardiotoxicidade por Antraciclinas: Revisão Sistemática e Metanálise
Source: Arq Bras Cardiol. 2023 May 17;120(5):e20220298. [Article in Portuguese] doi: 10.36660/abc.20220298 (PMC10484562; doi:10.36660/abc.20220298)
Supplement: Supplementary file 1 [file 2022-0298_AO_SupplementMaterialmeta-analysis.pdf]

Supplement Material

Table. PRISMA Checklist

Section and Topic	Item #	Checklist item	Location where item is reported
**TITLE**			
Title	1	Identify the report as a systematic review.	1
**ABSTRACT**			
Abstract	2	See the PRISMA 2020 for Abstracts checklist.	2
**INTRODUCTION**			
Rationale	3	Describe the rationale for the review in the context of existing knowledge.	4
Objectives	4	Provide an explicit statement of the objective(s) or question(s) the review addresses.	5
**METHODS**			
Eligibility criteria	5	Specify the inclusion and exclusion criteria for the review and how studies were grouped for the syntheses.	6
Information sources	6	Specify all databases, registers, websites, organisations, reference lists and other sources searched or consulted to identify studies. Specify the date when each source was last searched or consulted.	6
Search strategy	7	Present the full search strategies for all databases, registers and websites, including any filters and limits used.	6
Selection process	8	Specify the methods used to decide whether a study met the inclusion criteria of the review, including how many reviewers screened each record and each report retrieved, whether they worked independently, and if applicable, details of automation tools used in the process.	6
Data collection process	9	Specify the methods used to collect data from reports, including how many reviewers collected data from each report, whether they worked independently, any processes for obtaining or confirming data from study investigators, and if applicable, details of automation tools used in the process.	6
Data items	10a	List and define all outcomes for which data were sought. Specify whether all results that were compatible with each outcome domain in each study were sought (e.g. for all measures, time points, analyses), and if not, the methods used to decide which results to collect.	6

Section and Topic	Item #	Checklist item	Location where item is reported
	10b	List and define all other variables for which data were sought (e.g. participant and intervention characteristics, funding sources). Describe any assumptions made about any missing or unclear information.	6
Study risk of bias assessment	11	Specify the methods used to assess risk of bias in the included studies, including details of the tool(s) used, how many reviewers assessed each study and whether they worked independently, and if applicable, details of automation tools used in the process.	7
Effect measures	12	Specify for each outcome the effect measure(s) (e.g. risk ratio, mean difference) used in the synthesis or presentation of results.	7
Synthesis methods	13a	Describe the processes used to decide which studies were eligible for each synthesis (e.g. tabulating the study intervention characteristics and comparing against the planned groups for each synthesis (item #5)).	7
	13b	Describe any methods required to prepare the data for presentation or synthesis, such as handling of missing summary statistics, or data conversions.	7
	13c	Describe any methods used to tabulate or visually display results of individual studies and syntheses.	7
	13d	Describe any methods used to synthesize results and provide a rationale for the choice(s). If meta-analysis was performed, describe the model(s), method(s) to identify the presence and extent of statistical heterogeneity, and software package(s) used.	7
	13e	Describe any methods used to explore possible causes of heterogeneity among study results (e.g. subgroup analysis, meta-regression).	7
	13f	Describe any sensitivity analyses conducted to assess robustness of the synthesized results.	7
Reporting bias assessment	14	Describe any methods used to assess risk of bias due to missing results in a synthesis (arising from reporting biases).	7
Certainty assessment	15	Describe any methods used to assess certainty (or confidence) in the body of evidence for an outcome.	7
**RESULTS**			
Study selection	16a	Describe the results of the search and selection process, from the number of records identified in the search to the number of studies included in the review, ideally using a flow diagram.	8
	16b	Cite studies that might appear to meet the inclusion criteria, but which were excluded, and explain why they were excluded.	8
Study characteristics	17	Cite each included study and present its characteristics.	8
Risk of bias in studies	18	Present assessments of risk of bias for each included study.	8
Results of individual studies	19	For all outcomes, present, for each study: (a) summary statistics for each group (where appropriate) and (b) an effect estimate and its precision (e.g. confidence/credible interval), ideally using structured tables or plots.	9
Results of syntheses	20a	For each synthesis, briefly summarise the characteristics and risk of bias among contributing studies.	9
	20b	Present results of all statistical syntheses conducted. If meta-analysis was done, present for each the summary estimate and its precision (e.g.	

Section and Topic	Item #	Checklist item	Location where item is reported
		confidence/credible interval) and measures of statistical heterogeneity. If comparing groups, describe the direction of the effect.	
	20c	Present results of all investigations of possible causes of heterogeneity among study results.	9
	20d	Present results of all sensitivity analyses conducted to assess the robustness of the synthesized results.	9
Reporting biases	21	Present assessments of risk of bias due to missing results (arising from reporting biases) for each synthesis assessed.	9
Certainty of evidence	22	Present assessments of certainty (or confidence) in the body of evidence for each outcome assessed.	9
**DISCUSSION**			
Discussion	23a	Provide a general interpretation of the results in the context of other evidence.	9
	23b	Discuss any limitations of the evidence included in the review.	12
	23c	Discuss any limitations of the review processes used.	12
	23d	Discuss implications of the results for practice, policy, and future research.	11
**OTHER INFORMATION**			
Registration and protocol	24a	Provide registration information for the review, including register name and registration number, or state that the review was not registered.	2
	24b	Indicate where the review protocol can be accessed, or state that a protocol was not prepared.	5
	24c	Describe and explain any amendments to information provided at registration or in the protocol.	5
Support	25	Describe sources of financial or non-financial support for the review, and the role of the funders or sponsors in the review.	13
Competing interests	26	Declare any competing interests of review authors.	13
Availability of data, code and other materials	27	Report which of the following are publicly available and where they can be found: template data collection forms; data extracted from included studies; data used for all analyses; analytic code; any other materials used in the review.	6

Search Strategy for the Meta-analysis

We searched all databases using both controlled vocabulary (namely MeSH in MEDLINE and EMTREE in Embase) and free-text terms: ((“candesartan”) OR (“metoprolol”) OR (“spironolactone”) OR (“carvedilol”) OR (“enalapril”) OR (“telmisartan”) OR (“nebivolol”) OR (“statin”) OR (“dexrazoxane”) OR (“valsartan”) OR (“perindopril”) OR (“lisinopril”) OR (“bisoprolol”) OR (“labetalol”) OR (“atenolol”) OR (“propranolol”) OR (“?blocker”) OR (“ACE inhibitors”) OR (“angiotensin II receptor blocker”) OR (“angiotensin antagonists”) OR (“N-acetylcysteine”) OR (“Co-Q10”) OR (“prenylamine”)) AND ((“echocardiogram”) OR (“echocardiography”) OR (“magnetic resonance image”) OR (“left ventricular ejection fraction”) OR (“BNP”) OR (“troponin”) OR (“diastolic dysfunction”) OR (“LVEF”) OR (“strain”) OR (“biomarkers”) OR (“NT-proBNP”) OR (“cardiac events”) OR (“cardiac dysfunction”) OR (“LVEDD”) OR (“heart failure”) OR (“diastolic diameter”) OR (“systolic diameter”) OR (“brain natriuretic peptide”)) AND ((“anthracyclines”) OR (“trastuzumab”) OR (“chemotherapy”) OR (“cardiotoxicity”) OR (“doxorubicin”) OR (“epirubicin”) OR (“cumulative dose”) OR (“HER2”) OR (“taxane”) OR (“cancer”) OR (“breast cancer”) OR (“malignancies”) OR (“malignant homeopathies”) OR (“anthracyclines-induced”) OR (“global longitudinal strain”) OR (“lymphoma”) OR (“sarcoma”) OR (“antineoplastic agents”) OR (“radiotherapy”) OR (“cardiac toxicity”))

Table. Description of outcomes of the trials and inclusion/exclusion criteria

Study	Year	Primary outcome	Inclusion Criteria	Exclusion criteria
Kalay	2006	Systolic Function	Malignancy and planed anthracycline treatment	Earlier chemotherapy or radiotherapy, heart failure symptoms, dilated or restrictive CMP, coronary arterial disease history, moderate or severe mitral or aortic valve disease in baseline echocardiograph, any contraindication to carvedilol, bundle branch block, thyroid function disorder, or another comorbid disease. Use of angiotensin-converting enzyme inhibitors, angiotensin receptor blockers, diuretics, or beta blockers
Cardinale	2006	Occurrence of cardiotoxicity in the 2 groups, defined as an absolute decrease 10 percent units in rest LVEF associated with a decline below the normal limit value (50%)	Cancer patients undergoing high-dose chemotherapy	Presence of ischemic, valvular, and hypertensive heart disease; uncontrolled hypertension; LVEF <50%; age $\geq$ 65 years; and abnormal renal or hepatic functions. Additional exclusion criteria were intolerance or contraindication to ACEIs; ongoing therapy with -blocking agents, ACEIs, and angiotensin II receptors blockers; and systolic blood pressure <90 mm Hg. Patients developing acute (<2 weeks) cardiotoxicity after chemotherapy also were excluded
Georgakopoulos	2010	Incidence of cardiotoxicity	Patients with a diagnosis of lymphoma who were under treatment with anthracyclines	na
Salehi	2011	Incidence of cardiomyopathy	Patients with a diagnosis of breast malignancies and lymphoma who were under treatment with anthracyclines	Patients with records of chemotherapy, radiotherapy, symptoms of hyperemia, and cardiac insufficiency; patients with approved restrictive and hyperemia cardiomyopathy; patients with coronary vessel disease; those with moderate to severe insufficiency of the mitral and aortic valves in early echocardiography; patients with ramous blocks; patients with thyroid dysfunction; patients in whom carvedilol was contraindicated; patients with serious concurrent disease; patients taking angiotensin-converting enzyme inhibitors, angiotensin receptor blockers, diuretics, or beta-blockers; and patients under 12 years of age.
Dessi	2011	Prevention in the systolic function	Patients 18-70 years of age; an echocardiographic left ventricle ejection fraction (LVEF) value ≥55%; an SR value in the normal range (1.7-2.1 cm/ sec); an Eastern Cooperative Oncology Group (ECOG) performance status score of 0-2 (11); normal hepatic and renal function (bilirubin ≤1.5 mg/dl and creatinine ≤2.0 mg/dl); no concomitant medications known to interfere with inflammatory and oxidative stress parameters	History of cardiac disease, hypertension, diabetes and/or had been previously treated with mediastinal radiotherapy

Kaya	2013	Prevention of antracycline-induced cardiotoxicity in breast cancer patients receiving anthracycline-based chemotherapy	Female, breast cancer and planned antracycline-based chemotherapy	Presence of cardiomyopathy (dilated, restrictive or hypertrophic) detected by baseline echocardiography, coronary heart disease, moderate or severe aortic and/or mitral valve disease, prior chemotherapy or radiotherapy, alcohol abuse, any contraindications to nebivolol, bundle branch block, atrial fibrillation and dyslipidemia. Patients on other cardiac medications such as angiotensin-converting enzyme inhibitors, angiotensin receptor blockers, non-dihydropyridine calcium channel blockers, diuretics, statins or beta-blockers were also excluded
Bosch/ OVERCOME	2013	Change from baseline in global LVEF	Adult patients from 18 to 70 years old, in sinus rhythm and with normal echocardiographic LV ejection fraction (LVEF 50%), recently diagnosed with acute leukemia and referred for immediate intensive chemotherapy, and patients with relapsed or refractory Hodgkin and non-Hodgkin lymphoma and multiple myeloma undergoing autologous hematopoietic stem cell transplantation	Presence of congestive heart failure; LVEF < 50%; prior myocardial infarction or documented coronary artery disease; significant valvulopathy or myocardiopathy; renal failure (defined as an estimated glomerular filtration rate of < 30 ml/h/m2); hepatocellular insufficiency or grade III to IV increase of liver enzymes not secondary to tumoral liver infiltration; ongoing or expected need to be treated with ACEI, angiotensin II receptor blockers (ARB), or beta-blockers; prior allergy to ACEI or ARB; systolic blood pressure (SBP) lower than 90 mm Hg; asthma; atrioventricular block or sinus bradycardia (heart rate lower than 60 beats/min); persistent atrial fibrillation; need to be treated with a class I antiarrhythmic drug; pregnancy; and inability or unwillingness to give informed consent
Elitok	2014	preventive effect of carvedilol using strain imaging in patients with breast cancer treated with ANT	Female patients with breast cancer and anticipated anthracycline therapy,	Previous chemotherapy or radiotherapy; cardioprotective drug use, such as angiotensin-converting enzyme inhibitors, angiotensin receptor blockers, calcium channel blockers, statins, aldosterone receptor antagonist, and other beta-blockers; the presence of any cardiac disease detected upon baseline evaluation and echocardiography; and a history of chronic disease, such as diabetes, hypertension, chronic renal or hepatic failure, and any contraindication to carvedilol use
Akpek	2015	prevention of antracycline-induced cardiotoxicity	Female diagnosed with breast cancer and underwent an anthracycline chemotherapy protoco	Prior breast cancer and/or prior anthracycline exposure history; LVEF 2 mg/dL; presence of chronic kidney failure; potassium >5.3 mg/dL; presence of adrenal gland diseases; presence of severe liver failure; and co-morbidities such as coronary heart disease, hypertension, AF, and valvular heart disease
Gulati/ PRADA	2016	Change in left ventricular ejection fraction, as assessed by cardiac MRI	Women aged 18-70 years<br>Eastern Cooperative Oncology Group (ECOG) performance status 0-1<br>Serum creatinine < 140 µmol/L or estimated creatinine clearance > 60 ml/min<br>Systolic blood pressure >= 110 mgHg and < 170 mmHg<br>LVEF >= 50%	Hypotension, Bradycardia, Prior anthracycline chemotherapy regimen, Prior malignancy requiring chemotherapy or radiotherapy, Symptomatic heart failure, Systolic dysfunction (LVEF < 50%), Clinically significant coronary artery disease, valvular heart disease, significant arrhythmias, or conduction delays, Uncontrolled arterial hypertension defined as systolic blood pressure > 170 mm Hg, Treatment with ACEI, ARB or beta-blocker within the last 4 weeks prior to study start, Intolerance to ACEI, ARB or beta-blocker, Uncontrolled concomitant serious illness, Pregnancy or breastfeeding, Active abuse of drugs or alcohol, Suspected poor compliance, Inability to tolerate the MRI scanning protocol
Jhorawat	2016	Protective effect of carvedilol in Adriamycin -	Diagnosed with lymphoreticular malignancy and planned for	Earlier CT or thoracic radiotherapy, coronary arterial disease or established dilated

		induced cardiomyopathy	chemotherapy with regimen containing anthracycline	or restrictive CMP, moderate-to-severe valvular dysfunction or pericardial effusion, diabetes mellitus, renal dysfunction and thyroid disorder. Any contraindication to carvedilol, severe hepatic impairment and intake of other drugs that affected cardiac functions, for example, angiotensin converting enzyme (ACE)-inhibitor, angiotensin receptor blockers, diuretics or β-blockers, statins and antioxidants
Beheshti	2016	Evaluate the possible cardioprotective effects of carvedilol in patients with nonmetastatic breast cancer receiving doxorubicin, by means of measuring and comparing the strain and strain-rate parameters prior to and after chemotherapy	Nonmetastatic breast cancer, nonmenopausal women, no previous cardiac conditions (including ischemic heart disease, prolonged hypertension, and clinically important congenital or acquired valvular and myocardial diseases) or diabetes, no previous chemo/radiotherapy, taking no cardiac-related drugs and not having other cancers	New-onset cardiac symptoms or atrial fibrillation, unsatisfactory echocardiography and lack of patient compliance
Nabati	2017	Changes in LVEF	Adults aged 21–69 year with sinus rhythm, preserved left ventricular ejection fraction and normal hepatic, renal, and hematological function. All patients had newly diagnosed breast cancer	Previous chemotherapy or radiotherapy for another neoplasia, presence of CHF or cardiomyopathy, left ventricular ejection fraction (LVEF) ,50%, prior myocardial infarction or documented coronary artery disease, significant valvular regurgitation or stenosis, ongoing therapy with other drugs that may affect heart function, any contraindication to carvedilol, bundle branch block, systolic blood pressure less than 90 mm Hg, and pregnancy
Jambabai	2017	Change from baseline in LVEF measured	Age: 21–74 years and had an eastern cooperative oncology group (ECOG) performance status >2, normal sinus rhythm, and preserved left ventricular ejection fraction at baseline echocardiography. The patients had a newly diagnosed malignancy and adequate hematological, hepatic, and renal function	Previous chemotherapy or radiotherapy, presence of HF symptoms or documented dilated, hypertrophic or restrictive CMP, LV ejection fraction (LVEF)<50 %, prior myocardial infarction or documented coronary artery disease, presence of moderate or more mitral or aortic valve disease at baseline, persistent hypertension treated with angiotensin-converting enzyme inhibitors, angiotensin II receptor blockers, history of allergy to ACEIs or ARB, systolic blood pressure less than 90 mmHg, pregnancy, and reluctance to participate in this study
Abuosa	2018	Change in LVEF	Cancer patients aged >16 years who were treated with doxorubicin	Patients with left ventricular ejection fraction (LVEF) < 50% before enrolment to the study, known cardiomyopathy or on therapy for heart failure, bronchial asthma that required regular daily beta-2 stimulant therapy, severe peripheral arterial disease, second or third degree heart block, severe valvular heart disease, earlier therapy with anthracycline derivatives, coronary artery disease, thyroid function disorder and patients who were on beta-blockers or angiotensin convertingenzyme inhibitors
Cochera	2018	Preventive effect on doxorubicin induced cardiotoxicity	Age >18 years; patient's signed agreement to participate in the study; HER2 negative breast cancer diagnosis, previously untreated, with an indication of chemotherapy according to standardized international protocols; LVEF ≥50%.	Pregnancy and breastfeeding; previous radiotherapy or prior therapy with cytostatics; the use of cardioprotective drugs – such as angiotensin converting enzyme inhibitors, statins, calcium channel blockers, angiotensin receptor blockers, anti-aldosterone, and other adrenergic beta-blockers; 4) presence of a diagnosed cardiopathy in the initial clinical and echocardiographic evaluation; 5) history of chronic diseases such as diabetes mellitus, hypertension, chronic kidney disease, chronic liver disease; 6) contraindications for the treatment of beta-adrenergic blocker; and 7) doxorubicin intolerance

Avila/ CECCY	2018	Drop in LVEF of at least 10% from baseline	Age of at least 18 years, diagnosis of invasive breast adenocarcinoma, with an indication for adjuvant or neoadjuvant anthracycline therapy	Impossibility of LV function evaluation, prior history of chemotherapy or radiation, HF symptoms, prior diagnosis of cardiomyopathy, coronary artery disease, moderate to severe mitral and aortic disease, use of angiotensin-converting enzyme inhibitors (ACEi), angiotensin receptor blockers (ARB), or β-blockers, contraindication to the use of β-blockers, and patients with HER2 expression

Table. Study quality of the trials

Study	Ramdom Sequence Generation	Allocation Concealment	Blinding Participants and Personnel	Blinding of Outcome Assessment	Incomplete Outcome Data	Selective Reporting	Other Bias
Kalay	Unclear	Unclear	High	High	Low	Low	Low
Cardinale	Low	Low	High	High	Low	high	Low
Georgakopoulos	Unclear	Unclear	High	High	Low	Low	Low
Salehi	Unclear	Unclear	Low	Low	Low	Low	Low
Dessi	Low	Low	Low	Low	Low	Low	Low
Kaya	Unclear	Unclear	Low	Low	Low	Low	Low
Bosch/OVERCOME	Low	Low	High	High	Low	Low	Low
Elitok	Low	Unclear	High	High	Low	Low	High
Akpek	Unclear	Unclear	Low	Low	Low	Low	Low
Gulati/PRADA	Low	Low	Low	Low	Low	Low	Low
Jhorawat	Low	Unclear	High	High	Low	Low	High
Beheshti	Low	Low	Low	Low	Low	Low	Low
Nabati	Unclear	Unclear	High	High	Low	Low	High
Jambabai	Unclear	Unclear	High	High	Low	Low	Low
Abuosa	Low	Low	Low	Low	Low	Low	Low
Cochera	Unclear	Unclear	High	High	Low	High	High
Avila/CECCY	Low	Low	Low	Low	Low	Low	Low

Table. Absolute numbers of heart failure patients and death described in the trial

Study	Year	HF ctl No.	HF drug No.	Death ctl No.	Death drug No.
Kalay	2006	1	5	4	1
Cardinale	2006	14	0	2	0
Georgakopoulos	2010	3	1	0	0
Georgakopoulos	2010	3	2	0	0
Salehi	2011	5	5	4	1
Salehi	2011	5	1	4	2
Dessi	2011	na	na	na	na
Kaya	2013	na	na	na	na
Bosch/Overcome	2013	7	4	8	3
Elitok	2014	na	na	0	0
Akpek	Akpek	na	na	na	na
Gulati PRADA	2016	0	0	na	na
Gulati PRADA	2016	0	0	na	na
Gulati PRADA	2016	0	0	na	na
Jhorawat	2016	3	1	5	6
Beheshti	2016	na	na	na	na
Nabati	2017	3	2	0	1
Jambabai	2017	0	0	0	0
Abuosa	2018	4	0	2	1
Abuosa	2018	4	1	2	4
Abuosa	2018	4	0	2	4
Cochera	2018	20	7	na	na
Avila CECCY	2018	1	0	2	2

Abreviations- HF- Heart Failure, Ctl – control, No. number of patients
