# Supplementary material for: Antagonistas do Sistema Renina-Angiotensina e Betabloqueadores na Prevenção da Cardiotoxicidade por Antraciclinas: Revisão Sistemática e Metanálise
Source: Arq Bras Cardiol. 2023 May 17;120(5):e20220298. [Article in Portuguese] doi: 10.36660/abc.20220298 (PMC10484562; doi:10.36660/abc.20220298)
Supplement: Translation

# Translation: Renin-angiotensin System Antagonists and Beta-blockers in Prevention of Anthracycline Cardiotoxicity: a Systematic Review and Meta-analysis

**Authors:** Monica Samuel Avila, Suellen Rodrigues Rangel Siqueira, Lucas Waldeck, Silvia Moreira Ayub-Ferreira, Richard Takx, Marcio Sommer Bittencourt, Edimar Alcides Bocchi

**Affiliations:** 1 Brazil Departamento de Insuficiência Cardíaca – Instituto do Coração (InCor) do Hospital das Clínicas da Faculdade de Medicina da Universidade de São Paulo, São Paulo, SP – Brazil; 2 Netherlands Departmento de Radiologia – University Medical Center Utrecht, Utrecht, the Netherlands; 3 Brazil Centro de Pesquisas Clínicas e Epidemiológicas – Hospital Universitário – Universidade de São Paulo, São Paulo, SP – Brazil

**Keywords:** Drug Therapy, Heart Failure, Angiotensin-Converting Enzyme Inhibitors, Mineralocorticoid, Receptor Antagonists Anthracyclines

## Abstract

**Background:**

The evidence supporting the use of renin-angiotensin-aldosterone system (RAAS) inhibitors and beta-blockers for the prevention of anthracycline-induced cardiomyopathy is controversial.

**Objective:**

We performed a meta-analysis to assess the effectiveness of these drugs in preventing cardiotoxicity.

**Methods:**

The meta-analysis included prospective, randomized studies in adults receiving anthracycline chemotherapy and compared the use of RAAS inhibitors or beta-blockers versus placebo with a follow-up of 6 to 18 months. The primary outcome was change in left ventricular ejection fraction (LVEF) during chemotherapy. Secondary outcomes were the incidence of heart failure, all-cause mortality, and changes in end-diastolic measurement. Heterogeneity was assessed by stratification and meta-regression. A significance level of p < 0.05 was adopted.

**Results:**

The search resulted in 17 studies, totaling 1,530 patients. The variation (delta) in LVEF was evaluated in 14 studies. Neurohormonal therapy was associated with a lower delta in pre- versus post-therapy LVEF (weighted mean difference 4.42 [95% confidence interval 2.3 to 6.6]) and higher final LVEF (p < 0.001). Treatment resulted in a lower incidence of heart failure (risk ratio 0.45 [95% confidence interval 0.3 to 0.7]). There was no effect on mortality (p = 0.3). For analysis of LVEF, substantial heterogeneity was documented, which was not explained by the variables explored in the study.

**Conclusion:**

The use of RAAS inhibitors and beta-blockers to prevent anthracycline-induced cardiotoxicity was associated with less pronounced reduction in LVEF, higher final LVEF, and lower incidence of heart failure. No changes in mortality were observed. (CRD PROSPERO 42019133615)

## Introduction

Cancer is one of the most important cause of death in the world.^1^ The incidence of survival in patients with cancer has improved over the last years, particularly due to the success of chemotherapy treatment.^2^ However, these patients’ prognosis remains limited due to treatment complications, such as cardiotoxicity of anthracyclines, resulting in heart failure.^3^

Several strategies for primary prevention of anthracycline-induced cardiotoxicity have been proposed. Prevention of anthracycline-induced cardiotoxicity involves approaches that minimize the exposure of the drug, resulting in lower risk of potential cardiotoxicity and the decision to initiate cardioprotective drugs. Use of cardiovascular drugs, such as angiotensin-converting-enzyme inhibitors (ACEI), angiotensin receptor blocker (ARB), mineralocorticoid receptor antagonist (MRA), and beta-blockers are based on few clinical trials with controversial results. Use of preventive treatment with ACEI, ARB, MRA, or beta-blocker therapy in patients under anthracyclines chemotherapy with low cardiovascular baseline risk remains uncertain, and no recommendation can be made at this time.^4^

There are few meta-analyses published evaluating neurohormonal antagonist therapies in preventing cardiotoxicity. Some studies included pediatric populations^5,6^ and other interventions such as statins, dexrazoxane, or N-acetylcysteine,^7-10^ whereas other studies include only beta-blockers^11-15^ or only renin-angiotensin-aldosterone system (RAAS) antagonists. ^16, 17^ Recently, Vaduganathan et al. published a meta-analysis evaluating ACEI, ARB, MRA, and beta-blockers in preventing chemotherapy-related cardiotoxicity, including anthracycline and trastuzumab.^18^ As established, the cardiotoxicity mechanisms of anthracyclines and anti-HER2 therapies are distinct, which could be a confounding factor for the real impact of neurohormonal antagonist prevention of cardiotoxicity.

In face of controversial evidence supporting the use of angiotensin system inhibitors and beta-blockers for primary prevention of anthracycline-induced cardiotoxicity alone, we performed a systematic review and meta-analysis to assess the efficacy of these agents as prophylactic drugs for early onset of cardiotoxicity.

## Methods

### Literature search

We followed the Preferred Reporting Items for Systematic Reviews and Meta-Analyses (PRISMA) statement, and the PRISMA checklist is presented in the Supplementary Material.^19^ Our prespecified study protocol was registered with the International Prospective Register of Systematic Reviews (PROSPERO CRD 42019133615). We systematically searched PubMed, EMBASE, ClinicalTrials.Gov, and the Cochrane Central Register of Controlled Trials for randomized controlled trials of cardioprotective drugs, such as beta-blockers, ACEI, ARB, and MRA, in patients under anthracycline chemotherapy to evaluate the efficacy of these drugs in preventing cardiotoxicity. The list of terms used in the search is shown in the Supplementary Material. We limited the search to articles in English. We additionally searched the references of all articles retrieved. We included all randomized controlled trials using cardioprotective drugs on the active arm (ACEI, beta-blocker, ARB, or MRA) compared with placebo or usual care, with follow-up from 6 to 18 months, that reported cardiac function evaluated by echocardiogram or cardiac magnetic resonance, cardiac diameters and/or clinical outcomes (death, heart failure). We excluded abstracts, studies with shorter follow-up, pediatric population, studies without control arm, and non-randomized studies. No patients were included, and all study data are anonymous; therefore, no ethics committee or institutional review board approval was needed.

### Data extraction and outcomes

Two investigators (M.S.A. and S.R.R.S.) independently abstracted data using a standardized form, including study characteristics (design, inclusion and exclusion criteria), characteristics of the intervention (cardioprotective drug), patient characteristics (age, sex, cardiac risk factors, malignancy), and outcomes. For the outcomes, we defined a priori the primary outcome of change (delta) in left ventricular ejection fraction (LVEF) from baseline to the end of study. Secondary outcomes defined a priori included all-cause deaths, heart failure, and changes (delta) in measurement of end diastolic diameter by echocardiography.

### Data synthesis

We performed a narrative synthesis of the findings from the included studies, including description of the type of treatment, population characteristics, outcomes, and intervention content. We provided summaries of intervention effects for each study by calculating odds ratios for dichotomous outcomes and weighted mean differences for continuous outcomes. For studies that did not report the longitudinal differences in the changes in echocardiographic parameters over time, we used the reported differences with standard deviations, standard errors, or confidence intervals. For the studies that did not report any of the measures of dispersion for the change (delta), the standard errors were derived from the standard deviation of the pre- and post-measurements, inputting the values of correlations between the pretest and posttest, based on the correlation derived from other studies in which we had access to individual patient level data to derive the coefficients. The description of outcomes of the trials and inclusion/exclusion criteria are detailed in the Supplementary Material.

### Stratification and sensitivity analysis

We expected that a large number of studies with different outcomes and interventions could result in a high heterogeneity. Thus, we performed all analysis using random effects models. Moreover, when the heterogeneity measured by χ^2^ test and the I^2^ were greater than 50%, indicating substantial heterogeneity, we performed additional analyses according to study quality, study date, type of drug used for treatment, anthracycline dose, and characteristics of patients included in the study. This analysis was performed using stratified meta-analyses for categorical predictors and meta-regression for continuous predictors. We also assessed evidence of publication bias using funnel plots. The statistical analysis was performed using Stata 17.0 (StataCorp, United States), and the level of significance was defined as p < 0.05.

### Quality assessment

Two investigators (M.S.A. and S.R.R.S.) independently assessed study quality using the Cochrane Collaboration’s tool for assessing risk of bias in randomized trials.^20^ Disagreements were resolved by consensus.

### Quality of trials

Two investigators (M.S.A. and S.R.R.S.) assessed study quality using Cochrane Risk of Bias Tool. In particular, the assessment considered: random sequence generation, allocation concealment, blinding of participants and personnel, blinding of outcome and assessment, data evaluation, and other bias. Study quality is detailed in the Supplementary Material.

## Results

The systematic search resulted in 355 potentially relevant articles. After removal of trials that did not meet the inclusion criteria, with fewer than 6 months of follow up, non-randomized trials, lack of placebo control, and pediatric study, 17 trials were included in analysis. The diagram for the study selection is shown in Figure 1. The included trials from 2006 to 2018 with 1530 patients had similar inclusion criteria except for the type of cancer, although breast cancer was the most frequent disease. The characteristics of the included studies and baseline patient demographics are present in Table 1. Seven trials were double blinded, whereas 3 were single blinded, and 7 were not blinded. The follow-up was 6 months in 13 clinical trials and 12 months in 3 trials. Ten of the studies tested the influence of beta-blockers (carvedilol, metoprolol, or nebivolol); two of them tested ACEI (enalapril); one tested ARB (telmisartan or candesartan); one evaluated aldosterone antagonism (spironolactone); two analyzed the association of ACEI and beta-blocker, and one tested the association of ARB and beta-blocker.

**Figure 1:** – Trial selection process for the systematic review.

**Table 1:** – Baseline characteristics of randomized trials. Studies that used more than one cardioprotective drug were dismembered for better analysis. All the studies adopted 5% statistical significance

Study	Year	CPT Drug	Fem ctl no.	Fem drug no.	Age ctl (years)+	Age drug (years)+	No. pts ctl	No. pts drug	Malignancy	ANT	HPTN clt no. (%)	HPTN drug no. (%)	DM clt no. (%)	DM drug no. (%)	HLP ctl no. (%)	HLP drug no. (%)	Smoker ctl no. (%)	Smoker drug no. (%)	Radiation ctl no. (%)	Radiation drug no. (%)	FU months
Kalay^21^	2006	CVDL	21	22	49	46.8	25	25	LMP, BC	DOX, EPI	NA	NA	NA	NA	NA	NA	NA	NA	0	0	6
Cardinale^22^	2006	ENLP	39	33	44	47	58	56	AL, BC, ES, HLMP, MLM, NHLMP	EPI, IDA, DAU	4 (7)	3 (5)	1 (2)	1 (2)	2 (3)	2(4)	NA	NA	18 (31)	19 (34)	12
Georgakopoulos^26^	2010	MTPL	19	20	49.1	51	40	42	HLMP, NHLMP	DOX	6 (15)	10 (24)	6 (15)	10 (24)	10 (25)	14 (33)	16 (40)	17 (40)	9 (23)	8 (19)	12
Georgakopoulos	2010	ENLP	19	21	49.1	47.4	40	43	HLMP, NHLMP	DOX	6 (15)	14 (33)	6 (15)	3 (7)	10 (25)	11 (26)	16 (40)	20 (46)	9 (23)	9 (21)	12
Salehi^27^	2011	CVDL *	14	32	43.5	43.5	22	44	LMP, BC	DOX, EPI	NA	NA	NA	NA	NA	NA	NA	NA	0	0	4
Dessì^28^	2011	TMST	18	19	53	52.9	24	25	NHLMP, BC, others	EPI	0	0	0	0	NA	NA	NA	NA	0	0	12
Kaya^29^	2013	NBVL	18	27	50.5	51.4	18	27	BC	DOX, EPI	4 (22)	6 (22)	2 (11)	2 (7)	NA	NA	NA	NA	5 (28)	7 (26)	6
Bosch/OVERCOME^30^	2013	ENLP, CVDL	21	18	50.9	49.7	45	45	AL, HLMP, NHLMP	IDA, DAU	8 (18)	6 (13)	3 (7)	7(16)	1 (2)	3 (7)	4 (9)	13(29)	4 (9)	12(27)	6
Elitok^31^	2014	CVDL	40	40	52.9	54.3	40	40	BC	DOX	0	0	0	0	NA	NA	NA	NA	NA	NA	6
Akpek^32^	2014	ESPL	40	43	50.6	50	40	43	BC	DOX, EPI	0	0	NA	NA	NA	NA	NA	NA	NA	NA	6
Gulati/PRADA^23^	2016	CDST, MTPL	32	32	50.8	50	32	32	BC	EPI	0	1 (3)	0	0	NA	NA	7 (22)	6 (20)	23 (72)	18 (60)	6
Gulati/PRADA	2016	CDST	32	33	50.8	51.7	32	33	BC	EPI	0	5 (16)	0	1 (3)	NA	NA	7 (22)	7 (22)	23 (72)	19 (60)	6
Gulati/PRADA	2016	MTPL	32	32	50.8	50.5	32	32	BC	EPI	0	2 (6)	0	1 (3)	NA	NA	7 (22)	5 (16)	23 (72)	22 (69)	6
Jhorawat^33^	2016	CVDL	9	4	38.7	43.89	27	27	NHLMP, HLMP, AL	DOX	NA	NA	0	0	NA	NA	NA	NA	NA	NA	6
Beheshti^34^	2016	CVDL	40	30	39.9	42	40	30	BC	DOX	0	0	0	0	NA	NA	NA	NA	0	0	6
Nabati^35^	2017	CVDL	45	46	47.1	47.5	45	46	BC	DOX	5 (12)	11 (27)	5 (12)	3 (7)	NA	NA	NA	NA	0	0	6
Janbabai^36^	2017	ENLP	31	33	47	47.7	35	34	BC, HLMP, others	DOX	4 (11)	6 (18)	5 (14)	3 (9)	3 (9)	4 (12)	NA	NA	0	0	6
Abuosa^37^	2018	CVDL**	29	83	40.4	46.1	38	116	BC, LMP, others	DOX	4 (10)	14 (12)	6 (16)	21 (18)	2 (5)	6 (5)	NA	NA	NA	NA	6
Cochera ^38^	2018	NBVL	30	30	52	53	30	30	BC	DOX	0	0	0	0	3 (10)	4 (13)	3 (10)	2 (6)	0	0	6
Avila/CECCY^24^	2018	CVDL	96	96	52.9	50.8	96	96	BC	DOX	9 (9)	3 (3)	5 (5)	4 (4)	2 (2)	6 (6)	26 (27)	24 (25)	0	0	6

HLP: hyperlipidemia; DM: diabetes melitus; HPTN: hypertention; Ctl: control; ANT: anthracycline; DOX: doxorubicin; EPI:epirubicin; DAU: daunorubicin; IDA: idarubicin; CPT: cardioprotective; LMP: linphoma; BC- breast cancer; AL: acute leukemia; ES: Ewing’s sarcoma; HLMP: Hodgkin’s lymphoma; MLM: myeloma; NHLMP: non-Hodgkin’s lymphoma; Pts: patients; Others: endometrium; salivary gland; ovary; lung cancer; Wilms tumor; Fem: female; FU: follow-up ; CVDL: carvedilol ; MTPL: metoprolol; NBVL: nebivolol; CDST: candesartan; ENLP: enalapril ; ESPL: espironolactone; TMST: telmisartan; No.: number of patients; NA: not applicable. + Age was expressed as median. * The study tested the doses of 12.5 mg and 25 mg.

All the 17 studies assessed left ventricular function, and 10 studies analyzed left ventricular end-diastolic diameter (LVEDD) for the detection of cardiotoxicity via echocardiography. Doxorubicin was the most common anthracycline chemotherapy included in the trials and the median cumulative dose (interquartile range) was 241 (240 to 369) mg/m^2^ in placebo group and 286 (254.2 to 383) in cardioprotective drug group.

### Absolute change in left ventricular ejection fraction

Fourteen studies analyzed the delta of ejection fraction. Values of LVEF and LVEDD from baseline to the end of the studies are summarized in Table 2. Pooled results showed that patients receiving beta-blockers and RAAS blockers had less prominent changes in LVEF than the control group (weighted mean difference of the delta in LVEF: 4.42, 95% confidence interval 2.27 to 6.57, p = 0.0001, Central Illustration). However, significant heterogeneity was observed, even after stratification by drug used in the treatment (I-squared = 92.7%), though effect sizes were comparable for all drugs. Additional meta-regressions using age, cumulative dose of anthracyclines, or year of the study were unable to identify any factor associated with the heterogeneity.

**Table 2:** – Changes in LVEF and LVEDD. Studies that used more than one cardioprotective drug were dismembered for better analysis

Study	Year	Cum. ANT dose ctl (mg/m^**2**^)	Cum. ANT Dose Drug (mg/m^**2**^)	LVEF baseline ctl (%)	LVEF baseline drug (%)	LVEF end of study ctl (%)	LVEF end of study drug (%)	LVEDD baseline ctl (mm)	LVEDD baseline drug (mm)	LVEDD final ctl (mm)	LVEDD final Drug (mm)
Kalay^21^	2006	513.6	525.3	69.7 + 7.3	70.6 + 8	52.3 + 14	69.7 + 6	45.6 + 5	47.6 + 5.6	50.9 + 5.6	47.4 + 3.7
Cardinale^22^	2006	338 + 167	332 + 191	62.8 + 3.4	61.9 + 2.9	51.9 + 7.9	61.3 + 3.9	NA	NA	NA	NA
Georgakopoulos^26^	2010	386.4 + 5.7	387.5 + 6.8	67.6 + 7.1	65.7 + 5	66.6 + 6.7	63.3 + 7.4	48 + 6	47 + 5	48 + 5	49 + 4
Georgakopoulos	2010	386.4 + 5.7	373.1 + 6.3	67.6 + 7.1	65.2 + 7.1	66.6 + 6.7	63.9 + 7.5	48 + 6	49 + 4	48 + 5	50 + 5
Salehi^27^Carvedilol 12.5 mg	2011	540.2 + 31.1	531.5 + 29.9	58.56 + 3.62	60.5 + 5.07	53.9 + 3.8	53.1 + 7.76	41.3 + 0.6	41.7 + 0.39	45.6 + 0.57	45 + 0.46
Salehi Carvedilol 25 mg	2011	540.2 + 31.1	521.14± 38.97	58.56 + 3.62	61 + 7.06	53.9 + 3.8	56.8 + 6.2	41.3 + 0.6	39.3 + 0.34	45.6 + 0.57	40.9 + 0.37
Dessì^28^	2011	400	400	66 + 5	66 + 7	65+ 7	68 + 4	NA	NA	NA	NA
Kaya^29^	2013	235 + 48	527 + 29	66.6 + 5	65.6 + 4.8	57.5 + 5.6	66.6 + 5.5	47.2 + 3.8	47 + 4.4	52 + 4.6	47.1 + 4
Bosch Overcome^30^	2013	241 + 162	290 + 189	62.59 + 5.38	61.67 + 5.11	59 + 6	62 + 5	NA	NA	NA	NA
Elitok^31^	2014	523.3	535.6	65 + 4.5	66 + 6.1	63.3 + 4.8	64.1 + 5.1	44.3 + 3.1	45 + 14.2	44.1 + 4.1	44.6 + 3.2
Akpek^32^	2014	394.2	430.2	67.7 + 6.3	67 + 6.1	53.6 + 6.8	65.7 + 7.4	46 + 5	46 + 4	52 + 4	49 + 4
Gulati PRADA^23^ candesartam +metoprolol	2016	301.3 + 75.57	297.3 + 72.5	63.6 + 4.1	62.2 + 4.4	60.3	61.1	NA	NA	NA	NA
Gulati PRADA canndesartan	2016	301.3 + 71.57	297.5 + 71.8	63.6 + 4.1	62.3 + 5.3	60.3	61.63	NA	NA	NA	NA
Gulati PRADA metoprolol	2016	301.3 + 75.57	301.3 + 72.5	63.6 + 4.1	63.5 + 5.0	60.3	60.8	NA	NA	NA	NA
Jhorawat^33^	2016	252.6 + 77.82	267.3 + 76.1	67.56+ 5.98	63.19+ 7.22	60.82+ 11.28	63.88+ 8.56	47.24+ 5.13	46.35+ 7.71	48.5 + 5.75	47.95 + 5.28
Beheshti^34^	2016	240	240	59.41+ 4.20	61.31+ 3.21	59.30 + 4.29	61.06 + 3.39	NA	NA	NA	NA
Nabati^35^	2017	359.9 + 27.1	348.5 + 34.8	61.13 + 4.97	58.72 + 4.69	51.67 + 6.01	57.44 + 7.52	NA	NA	NA	NA
Janbabai^36^	2017	266.6 + 21.7	363.3 + 34.8	59.61 + 5.70	59.39 + 6.95	46.31 + 7.04	59.93 + 7.83	NA	NA	NA	NA
Abuosa^37^Carvedilol 6.25 mg	2018	265.6 + 98.5	252 + 65	62.0 + 4.6	61.4 + 3.9	58.2 + 6.6	61.4 + 3.9	45.3 + 5.3	46.0 + 5.1	45.9 + 7.5	46.8 + 4.0
Abuosa Carvedilol 12.5 mg	2018	265.6 + 98.5	282 + 78	62.0 + 4.6	60.0 + 4.2	58.2 + 6.6	60.0 + 4.1	45.3 + 5.3	44.8 + 4.3	45.9 + 7.5	46.0 + 3.7
Abuosa Carvedilol 25 mg	2018	265.6 + 98.5	261 + 101	62.0 + 4.6	60.5 + 4.2	58.2 + 6.6	60.4 + 4.2	45.3 + 5.3	44.6 + 6.3	45.9 + 7.5	45.5 + 5.3
Cochera^38^	2018	519 + 9	521 + 6	61+ 2	62+ 4	60 + 3	61 + 3	44.8 + 4.2	45.1+ 4.2	46.1 + 3.5	46.2 + 2.9
Avila CECCY^24^	2018	240	240	65.2 + 3.6	64.8 + 4.7	63.9 + 5.2	63.9 + 3.8	44.9 + 3.6	44.1 + 3.3	46.4 + 4.0	45.2 + 3.2

Cum: cumulative; ANT: anthracycline; LVEF: left ventricular ejection fraction; HF: heart failure; Ctl: control; No.: number of patients; LVEDD: left ventricular end-diastolic diameter; NA: not applicable. + The evaluation of LVEF was performed by cardiac magnetic resonance. **The study tested the doses of 6.25 mg, 12.5 mg and 25 mg.

**Central Illustration:** : Renin-angiotensin System Antagonists and Beta-blockers in Prevention of Anthracycline Cardiotoxicity: a Systematic Review and Meta-analysis

Impact of cardioprotective drugs on left ventricular ejection fraction. CI: confidence interval; RAAS: renin-angiotensin-aldosterone system; RR: risk ratio.

### Heart failure and death

Twelve studies reported the influence of neurohormonal drugs and beta-blockers on the incidence of heart failure and eleven on death. However, after pooling the results from the twelve studies, the presence of cardioprotective drugs was associated with fewer symptoms of heart failure during and after anthracycline use (risk ratio 0.45, 95% confidence interval 0.28 to 0.72, p = 0.32, Figure 2). Heterogeneity between studies was not significant (I-squared = 11.18%), and no potential publication bias was identified. The absolute numbers of heart failure and death are reported in the Supplementary Material.

**Figure 2:** – Impact of cardioprotective drugs on occurrence of heart failure. CI: confidence interval; RAAS: renin-angiotensin-aldosterone system; WMD: weighted mean difference.

## Discussion

The present meta-analysis analyzed the protective effects of RAAS inhibitors and beta-blockers against anthracycline-induced cardiotoxicity. We selected 17 randomized trials and found a benefit of cardioprotective agents on changes in LVEF and symptoms of heart failure. Neurohormonal therapy was associated with a lower delta in LVEF and fewer symptoms of heart failure, and there was no effect on mortality. Despite the positive impact of neurohormonal drugs, we found a high heterogeneity between the studies; thus, interpretation of these findings needs to be contextualized, and a potential for publication bias should be considered.

The field of cardio-oncology has been studied extensively in the last 15 years. Kalay et al.^21^ showed, in 2006, that use of carvedilol could prevent the decrease in ejection fraction and the increase in left ventricle diameters in patients using anthracyclines, without a significant change in mortality. In another important trial, Cardinale et al.^22^ showed that the use of ACEI could reduce the elevation in left ventricular systolic diameter and prevent cardiotoxicity, in patients who had higher troponin changes after the chemotherapy cycle.

More recently, the PRADA trial, a randomized, placebo-controlled study, evaluated use of candesartan, metoprolol, and combined use of both drugs in primary prevention of anthracycline cardiotoxicity. The study found benefit only with candesartan, demonstrating a smaller extracellular volume assessed by magnetic resonance imaging and attenuated reduction in LVEF.^23^ The most recent randomized trial published, the CECCY Trial, was a single-center, randomized trial that tested carvedilol as a cardiac protector in breast cancer patients undergoing chemotherapy with anthracyclines. It showed no significant difference in ventricular dysfunction, but it did show a benefit in left ventricular diastolic diameter and troponin in the carvedilol group.^24^

The analysis of the outcome of heart failure performed individually in each study showed no statistical difference. However, when we analyzed the total population of all studies, we observed a better outcome in the group using beta-blockers and RAAS inhibitors, with significant results.

Heterogeneity differs between the analyzed outcomes. We observed significant heterogeneity in the assessment of the ejection fraction delta, which potentially reflects variation in study population due to differences in cardioprotective therapy, malignancy, and doses of anthracyclines. Regarding evaluation of the clinical outcome, we observed a low heterogeneity.

Some meta-analyses evaluated the impact of neurohormonal therapy on anthracycline-induced cardiotoxicity. Kheiri et al. evaluated the impact of carvedilol on the prevention of anthracycline-induced cardiotoxicity and demonstrated a possible benefit attenuating the decrease in the LVEF. However, this study did not include RAAS inhibitors.^11^ Recently Caspani et al. published a meta-analysis that evaluated neurohormonal therapies in this scenario, including a smaller number of trials and sample size. They found benefit in preventing LVEF reduction in the drug arm, but did not find an impact of cardioprotective drug on heart failure.^25^ Vaduganathan et al. published a meta-analysis evaluating ACEI, ARB, MRA, and beta-blockers in preventing chemotherapy-related cardiotoxicity, including, anthracycline and trastuzumab. The authors concluded that neurohormonal therapies had a positive impact on reducing decline in left ventricular function with a high heterogeneity, which is consistent with our analysis. Nonetheless, the inclusion of a trial with trastuzumab could be a confounding factor, as the cardiotoxicity mechanism is different from anthracycline.^18^

Our results reveal the need for studies with larger populations, with higher potential to show the real benefit of cardioprotective drugs in cardiotoxicity.

### Study limitations

Our meta-analysis has several important limitations. The majority of the studies included evaluated LVEF using standard echocardiography, and few included left ventricular structure. Changes in LVEF are a very heterogeneous measurement, and inter-observer variability was not reported in all trials. Concerning the endpoint of heart failure, there are missing data in some articles, and this could compromise the results of this outcome. Regarding anthracycline dose, some articles reported total dose of anthracycline and did not report mg/m^2^ dose. Moreover, as the studies included different anthracyclines, the doses were different between the trials. The limited sample sizes in some trials and missing data on cardiovascular risk factors could prevent subgroup analyses by cardiovascular risk.

## Conclusion

We conclude that RAAS antagonists and beta-blockers for prevention of anthracycline-induced cardiotoxicity were associated with less pronounced reduction in LVEF, higher final LVEF, and lower incidence of heart failure. No changes in mortality were observed. Significant heterogeneity was observed across the studies in the assessment of the ejection fraction delta, which potentially reflects variation in study population. It is necessary to conduct further studies with larger populations, with consistent and significant results demonstrating the benefit of cardioprotective drugs in cardiotoxicity.

## *Supplemental Materials

For additional information, please click here.
